# Supplementary figures and images for: Children, caregivers and health workers’ perceptions and experiences of the XTEMP-R tool to improve tuberculosis treatment
Source: PLOS Glob Public Health. 2025 Oct 8;5(10):e0005269. doi: 10.1371/journal.pgph.0005269 (PMC12507288; doi:10.1371/journal.pgph.0005269)

## S1 Fig: Description of how to use the XTEMP-R® tool


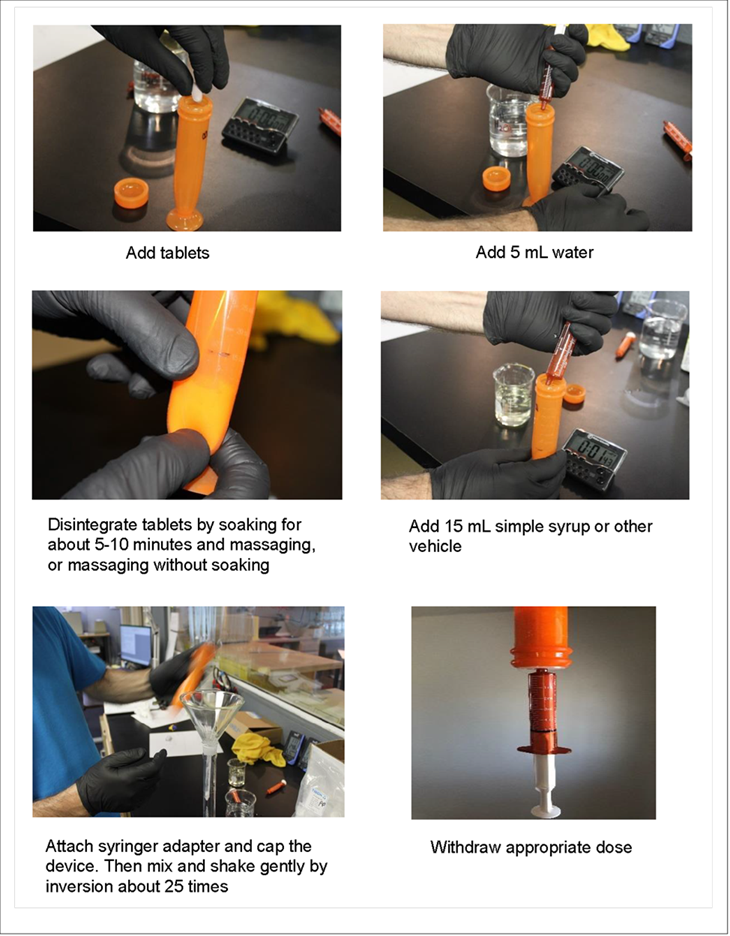

Supplement: S1 Fig — (DOCX) [file pgph.0005269.s001.docx]
