# Supplementary material for: Children, caregivers and health workers’ perceptions and experiences of the XTEMP-R tool to improve tuberculosis treatment
Source: PLOS Glob Public Health. 2025 Oct 8;5(10):e0005269. doi: 10.1371/journal.pgph.0005269 (PMC12507288; doi:10.1371/journal.pgph.0005269)
Supplement: S1 File — (DOCX) [file pgph.0005269.s002.docx]

## S1 File:

<https://www.tballiance.org/xtemp-videos/>
