# Supplementary material for: Children, caregivers and health workers’ perceptions and experiences of the XTEMP-R tool to improve tuberculosis treatment
Source: PLOS Glob Public Health. 2025 Oct 8;5(10):e0005269. doi: 10.1371/journal.pgph.0005269 (PMC12507288; doi:10.1371/journal.pgph.0005269)
Supplement: Attachment — Submitted filename: Comments for Manuscript Number PGPH-D-25-01547.docx [file pgph.0005269.s003.docx]

**Reviewer Recommendation and Comments for Manuscript Number PGPH-D-25-01547**

Obviously, a lot of work has gone into this and I commend the authors. However, there are a few things authors may consider:

| s/n | Comments | Recommendations |
| --- | --- | --- |
| 1 | Purposeful sampling; especially with the caregivers | Provide justification for the sampling technique and how you minimized bias |
| 2 | Unclear how authors determined the sample size | Include information about sample size determination |
| 3 | Good mention of the consent process, but the healthcare workers (HCWs) were not mentioned | Include consent process for HCWs for completeness |
| 4 | It is unclear what additional information the Caregivers in component 1 provided that could not have been collected as baseline from participants in component 2 | Include justification for the choice of participants in both components |
| 5 | Use of AI or any software | Mention if any software or AI was used in data collection, transcription, etc. |
