# Supplementary material for: Children, caregivers and health workers’ perceptions and experiences of the XTEMP-R tool to improve tuberculosis treatment
Source: PLOS Glob Public Health. 2025 Oct 8;5(10):e0005269. doi: 10.1371/journal.pgph.0005269 (PMC12507288; doi:10.1371/journal.pgph.0005269)
Supplement: Attachment — Submitted filename: 20250907_Response to Reviewers_PGPH-D-25-01547.docx [file pgph.0005269.s006.docx]

**Response to Reviewers for Manuscript: PGPH-D-25-01547**

The editor,

Thank you for allowing us the opportunity to clarify some points in the manuscript and to bring it in line with the journal’s specifications.

## Response to Reviewer #1:

We would like to thank Reviewer 1 for their comments and suggestions.

|  | Comments | Changes |
| --- | --- | --- |
| 1 | Purposeful sampling; especially with the caregivers. Provide justification for the sampling technique and how you minimized bias | Thank you for this important suggestion:  We have added the following sentences to provide further clarity:  In the Methods section (lines: 135-138):  *“We used maximal variation sampling to purposively recruit caregivers whose children had a diverse range of DR-TB experiences across key variables, including their child’s age, sex, and language (as a proxy for ethnicity). In this way, we sought a heterogenous sample to improve the transferability of our findings.”*  We have also included the following reference to support this statement:  Tracy, S. J. (2010). Qualitative Quality: Eight “big-tent” criteria for excellent qualitative research. Qualitative Inquiry, 16(10), 837–851.  And Lines 148-150:  *“As in component one we used maximal variation sampling to purposively sample caregiver and child participants to maximise diversity in child age, sex and language.”*  In the Discussion section (lines 453-456):  *“In addition, our purposive recruitment strategy may have inadvertently favoured caregivers who were already more engaged with healthcare services, as they were involved in prior or ongoing studies. Therefore, this work may not fully detail the challenges experienced by caregivers in the wider population, particularly those who face significant barriers to accessing care.”* |
| 2 | Unclear how authors determined the sample size. Include information about sample size determination. | We have added the following sentence to the Method section (Line 163-165):  *“Data collection and analysis was iterative and ongoing, and involved comparing each interview and emergent codes with previously collected data. Data collection and analysis continued until we reached code saturation – the point at which new data collection was not yield new insights.”*  We have also included the following reference to support this statement:  Hennink, M.M., Kaiser, B.N., and Marconi, V.C. Code Saturation Versus Meaning Saturation: How Many Interviews Are Enough? Qual Health Res 2017; 27(4): 591–608. |
| 3 | Good mention of the consent process, but the healthcare workers (HCWs) were not mentioned. Include consent process for HCWs for completeness. | Apologies for this oversight. We have added the following sentence to the Ethics section (Lines 177-179):  *“Healthcare workers provided written informed consent prior to participation, after permission to approach them was obtained from site and healthcare facility managers.”* |
| 4 | It is unclear what additional information the Caregivers in component 1 provided that could not have been collected as baseline from participants in component 2. Include justification for the choice of participants in both components. | Thank you for this insightful question. We have provided the following clarifying statements in the Methods section.  Lines 138-140:  *“Findings from Component One informed our initial interactions with caregivers and children in Component Two, especially regarding managing expectations and providing clear instructions for the XTEMP-R® tool.”*  Lines 143-146:  *“Through Component two we sought to (a) understand how caregivers and their children used the XTEMP-R® in their everyday treatment routine, and (b) inform refinement of the XTEMP-R® tool prototype, including how its use is explained to future potential users.”* |
| 5 | Use of AI or any software. Mention if any software or AI was used in data collection, transcription, etc. | We did not use any artificial intelligence or automated software tools for data collection, transcription, or analysis. Therefore, a statement to this effect was not included in the manuscript. |
